# From crisis to recovery: A case report on nursing strategies for hepatitis E post-cardiac arrest

**DOI:** 10.1097/MD.0000000000044325

**Published:** 2025-09-05

**Authors:** Rong Xu, Junjun Wu, Lili Dong, Fang Ding, Wenli Wu, Su Zheng

**Affiliations:** a Department of Gastroenterology, Hangzhou Third People’s Hospital, Hangzhou, Zhejiang, China; b Nutrition Department, Hangzhou Third People’s Hospital, Hangzhou, Zhejiang, China.

**Keywords:** acute hepatitis E, ECMO, gut-liver axis, nursing intervention, nutritional support, transfusion-associated infection

## Abstract

**Rationale:**

Extracorporeal membrane oxygenation (ECMO) is a life-support technology for refractory cardiac arrest, but the massive blood transfusions required during treatment significantly increase the risk of transfusion-related infections. Hepatitis E virus (HEV) – traditionally linked to fecal-oral transmission – is increasingly recognized as a transfusion-transmitted pathogen, especially in emergency settings where urgent blood product infusion is common and routine HEV screening in blood banks is often lacking. However, nursing strategies for managing acute HEV infection after ECMO remain poorly defined, highlighting the need to address this clinical gap.

**Patient concerns:**

A 35-year-old female nurse developed sudden cardiac arrest due to idiopathic ventricular fibrillation and underwent ECMO. Post-ECMO, she received red blood cells and plasma transfusions. On postoperative day 15, she had worsening liver function (alanine aminotransferase 938 U/L, total bilirubin 69.3 μmol/L) and abnormal coagulation function (prothrombin time [PT] 14.5 seconds), along with intermittent low-grade fever (37.3–38.0°C); subsequent jaundice of the skin, sclera, and urine developed.

**Diagnoses:**

Next-generation sequencing confirmed acute HEV infection. The diagnosis was further supported by typical liver function abnormalities (marked elevation of transaminases and bilirubin), abnormal coagulation (PT 14.5 seconds), and clinical manifestations of HEV infection (fever, jaundice), with no evidence of other etiologies (e.g., viral hepatitis A/B/C, drug-induced liver injury).

**Interventions:**

Comprehensive nursing and clinical interventions were implemented. Daily monitoring: liver function (alanine aminotransferase, aspartate aminotransferase, bilirubin), coagulation status (with focus on PT, e.g., baseline PT 14.5 seconds), and jaundice-related symptoms (skin/sclera color, pruritus, urine color); gastrointestinal management: *Bacillus licheniformis* (0.5 g twice daily) to regulate intestinal flora, and lactulose (15 mL twice daily) to promote bowel movement, maintaining gut–liver axis balance; personalized nutritional support: Collaboration with the nutrition department to provide a low-fat semi-liquid diet (1500–1600 kcal/d, 75–80 g branched-chain amino acid-rich protein, and adequate vitamins/minerals); and cardiac follow-up: planning and implementation of implantable cardioverter defibrillator (ICD) implantation on postoperative day 50 (after resolution of liver injury and stabilization of coagulation function).

**Outcomes:**

After 49 days of hospitalization, the patient’s liver function normalized (total bilirubin within normal range, albumin increased from 31.3 to 35.1 g/L), coagulation function (PT) returned to normal, and jaundice resolved. She successfully underwent ICD implantation on postoperative day 50. A 3-month follow-up showed no chronic liver damage, and serum HEV-IgM turned negative at 6 months; no malignant arrhythmias or ICD discharges were recorded during follow-up.

**Lessons:**

This case emphasizes 3 key lessons: Firstly, for patients receiving ECMO and blood transfusions, close monitoring of liver function, coagulation indicators (e.g., PT), and clinical signs of HEV infection (fever, jaundice) is critical for early diagnosis; secondly, multimodal interventions – combining targeted monitoring (including coagulation tracking), gut–liver axis regulation, and personalized nutrition – are effective for managing acute HEV infection post-ECMO; and thirdly, timing of ICD implantation (e.g., postoperative day 50, after liver and coagulation stabilization) and collaboration between nursing teams, nutrition departments, and cardiac specialists ensure holistic care, supporting both liver recovery and long-term cardiac safety.

## 1. Introduction

Acute respiratory cardiac arrest is a serious clinical emergency, affecting over 6 million people worldwide each year.^[1]^ Its pathogenesis is complex and directly affects patient prognosis. As a salvage treatment, extracorporeal membrane oxygenation (ECMO) can provide temporary cardiopulmonary support to patients with refractory cardiac arrest, helping to stabilize hemodynamics.^[2]^ Nevertheless, the large number of blood transfusions required during ECMO treatment inevitably increases the risk of transfusion-related infectious diseases.

Acute Hepatitis E virus (HEV) infection has been on the rise worldwide in recent years, especially against the backdrop of transfusions related to infections. The positive rate of HEV RNA among European blood donors is approximately 0.01% to 0.076%.^[3]^ HEV infection poses a significant threat to the health of patients.^[4]^ The transmission routes of hepatitis E mainly include fecal-oral transmission, and in recent years, iatrogenic blood transfusion transmission has been gradually recognized. Transmission through blood transfusion is particularly prominent in emergency treatment, as emergency measures often involve the infusion of a large number of blood products, increasing the risk of infection.^[5]^ This article reports the nursing experience of a patient who received ECMO treatment after sudden death from idiopathic ventricular fibrillation and developed acute icteric hepatitis E after blood transfusion, with the aim of providing a reference for the nursing of similar cases. This case was approved by hospital ethics. (2025KA161)

## 2. Case report

### 2.1. Chief complaint

The chief complaints were sudden cardiac arrest 12 days after ECMO surgery and liver function deterioration 15 days later.

### 2.2. Disease description

The patient was a 35-year-old female who suddenly died and underwent cardiopulmonary resuscitation and tracheal intubation. The patient developed persistent ventricular tachycardia and ventricular fibrillation during cardiopulmonary resuscitation, and after multiple electrical cardioversion, ECMO was performed due to persistent cardiogenic shock. The ventilator was successfully weaned on the 6^th^ day after the operation. Postoperative secondary pulmonary infection was treated with piperacillin-tazobactam injection at 4.5 g Q12H combined with vancomycin injection at 1 g Q12H for anti-infection treatment for 10 days. The patient’s postoperative condition was stable, and she was transferred to a general ward on day 12. The liver function was close to normal 12 days after the operation.

**Figure 1.**
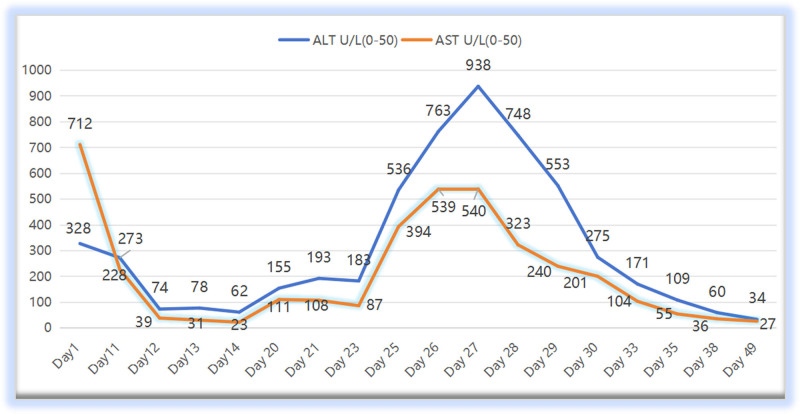
Alanine aminotransferase and AST levels during the onset of the disease. AST = aspartate aminotransferase.

However, 15 days postoperative, her liver function progressively worsened. Alanine aminotransferase (ALT) increased to 748 U/L, while total bilirubin increased to 37.1 μmol/L (Table 1, Fig. 1), with intermittent low-grade fever. The body temperature fluctuated between 37.3℃ to 38.0℃. Serum next-generation sequencing suggested HEV infection, and short paroxyventricular tachycardia was observed on the dynamic electrocardiogram reexamination 20 days after the operation. Considering the diagnosis of acute icteric hepatitis E, intravenous infusion of 150 mg qd of Tianqingganmei Injection, 465 mg qd of polyene phosphatidylcholine, and 1 g qd of adenosine disulfonate injection was administered for hepatoprotection and enzyme reduction. *Bacillus licheniensis* was used to regulate the intestinal flora, and lactulose was used for defecation, with close monitoring. Ribavirin was not used. Meanwhile, implantable cardioverter defibrillator (ICD) implantation was considered for idiopathic ventricular fibrillation.

**Table 1:** Indicators of liver function and coagulation function after the onset of the disease.

Laboratory paremeters	ALT U/L (0–50)	AST U/L (0–50)	Total bilirubin μmol/L (0–26.0)	Direct bilirubin μmol/L (0–8.0)	Albumin g/L (40.0–55.0)	Prothrombin time S (9.0–14.0)	Prealbumin mg/L (160–450)
Day1	328	712	–	–	–	–	–
Day11	273	228	19.2	–	–	–	–
Day12	74	39	–	–	–	11.1	–
Day13	78	31	11.8	4.7	32.1	–	192
Day14	62	23	12.8	5.3	30.7	–	186
Day 20	155	111	11.4	4.5	33.5	–	216
Day 21	193	108	9.3	3.7	34.4	–	212
Day 23	183	87	9.8	3.7	36.2	–	216
Day 25	536	394	14.9	7.1	34.8	–	186
Day 26	763	539	15.1	8.6	35.9	13.3	154
Day 27	938	540	37.1	29	32.5	14.1	135
Day 28	748	323	50.6	42.7	33	14.5	103
Day 29	553	240	61.2	52.6	33.2	13.7	88
Day 30	275	201	69.3	62.5	31.3	12.7	73
Day 33	171	104	63.6	58.7	30.9	–	77
Day 35	109	55	45.1	42	32.4	–	80
Day 38	60	36	26.4	23.4	35.1	10.5	103
Day 49	34	27	14.6	10.5	4	11	222

### 2.3. Nursing method

After being transferred to the gastroenterology ward, the patient was monitored daily for the color of her skin and sclera, skin itching, and urine coloration, and relevant records were made. The frequency of defecation and fatigue symptoms were also recorded. On postoperative day 22, the patient developed yellowing of the skin, sclera, and urine. The monitoring of indicators such as liver function and coagulation function was promptly initiated, and probiotics and laxative drugs were adjusted according to the defecation status. Nutritional guidance was provided in collaboration with the nutrition department (Table 2).

**Table 2:** Defecation status.

Postoperative Days	Bowel movement frequency	Bowel movement amount	Stool Color	Bristol stool scale
23	1	Normal	Brownish yellow	Type3
26	1	Normal	Brownish yellow	Type3
27	1	Less than normal	Yellowish brown	Type2
28	1	Normal	Yellowish green	Type3
29	1	Less than normal	Brownish yellow	Type3
30	1	Less than normal	Yellowish brown	Type2
31	0	/	/	/
32	6	More than normal	Yellowish brown	Type6, type7
33	0	/	/	/
35	1	Less than normal	Yellowish brown	Type5
38	2	Normal	Brownish yellow	Type4

### 2.4. Outcome

Following rigorous treatment and comprehensive nursing interventions, multiple rehabilitative parameters demonstrated favorable trends. By postoperative day 49, the patient achieved complete normalization of liver function, a critical marker of therapeutic efficacy. Total bilirubin levels declined from a peak of 69.3 μmol/L to within the reference range, accompanied by an elevation in serum albumin from 31.3 to 35.1 g/L – findings indicative of gradual recovery in hepatic bilirubin metabolism and synthetic capacity, which collectively sustain essential physiological homeostasis.

A 3-month follow-up assessment revealed no evidence of chronic liver injury, confirming that early therapeutic and nursing strategies effectively preserved hepatic integrity, preventing disease progression and chronic sequelae. At 6 months, serum HEV-IgM seroconverted to negative, signifying successful viral clearance by the immune system and resolution of acute hepatitis E virus infection.

The patient underwent uncomplicated implantation of an ICD. Subsequent surveillance documented no episodes of malignant arrhythmias or ICD discharges, reflecting stable cardiac function and validating the device’s role in mitigating risks of life-threatening events such as sudden cardiac arrest.

These outcomes collectively demonstrate that the integrated interventions not only ameliorated hepatic dysfunction and viral clearance but also stabilized cardiac status, underscoring the clinical value of multimodal management in this complex case.

### 2.5. History of blood transfusion

During ECMO treatment, 8.5 U of red blood cell suspension and 1810 mL of plasma products were transfused. The blood products passed routine screening (negative for HIV, HBV, HCV, and syphilis); however, HEV RNA detection was not performed, as HEV was not routinely screened in blood banks in this region.

### 2.6. Epidemiological investigation

The patient had no travel history to epidemic areas, had not consumed undercooked meat or shellfish, and had no clear history of contact with HEV.

## 3. Discussion

**Figure 2.**
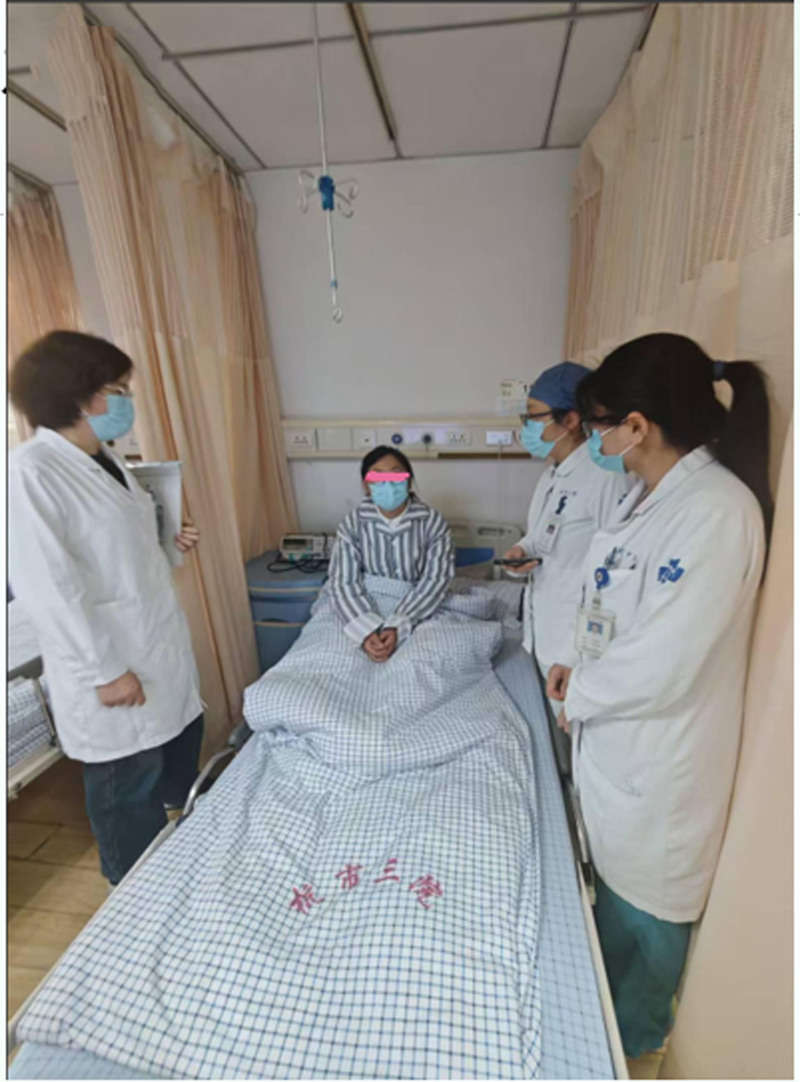
Nutritionists work alongside physicians and nurses to create personalized meal plans.

**Figure 3.**
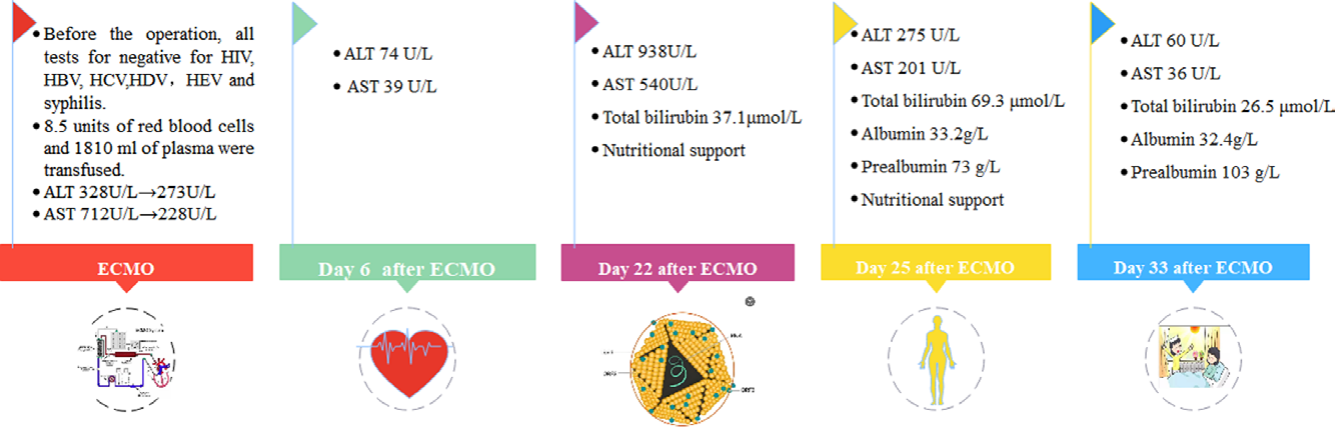
The timeline of hepatitis E infection after blood transfusion. ALT = alanine aminotransferase, AST = aspartate aminotransferase, ECMO = extracorporeal membrane oxygenation.

With the advancement of technology in recent years, ECOM has become a lifesaving measure for patients with sudden cardiac arrest experiencing continuous cardiogenic shock. When maintaining hemodynamic stability, red blood cell and/or plasma transfusion therapy is required according to the condition, which increases the risk of blood transfusion. In our case, the patient was successfully rescued. The hemodynamics were stable and the liver function was improving. As shown in Figure 2, the AST/ALT ratio was 2.17 after sudden cardiac arrest. Subsequently, 6 days after ECMO, the levels of liver enzymes returned to nearly normal. This indicates ischemic liver damage and is consistent with findings reported in previous studies.^[6,7]^ However, during the period of active anti-infection treatment, the patient still had low fever and liver damage worsened again, as evidenced by elevated liver enzymes and worsening jaundice. Drug-induced hepatitis, autoimmune hepatitis, and hepatitis A, B, and C viral factors were excluded again. Meanwhile, serum HEV-IgM antibodies and next-generation sequencing of serum samples indicated hepatitis E virus infection. The patient was hospitalized throughout the illness period; dietary factors were also excluded. It is concluded that hepatitis E virus infection is directly related to this blood transfusion (Fig. 3). Similar cases have also been reported in Hong Kong, China.^[8]^

### 3.1. Maintain the intestinal microecology and the balance of the liver-gut axis

The gut-liver axis is the core regulatory network for the occurrence and development of liver diseases, including 3 key links: the dysbiosis of the flora caused by intestinal barrier dysfunction; the transfer of microbial metabolites to the liver through the portal vein; the triggering of immune and metabolic disorders in the liver.^[9]^ Recent studies have shown that metabolites of the intestinal microbiota affect the pathological processes of the liver through multiple molecular pathways. Specifically, lipopolysaccharide (LPS) induces excessive secretion of pro-inflammatory factors TNF-α and IL-6 by activating the TLR4/MyD88/NF-κB signaling pathway in liver Kupffer cells. This mechanism has been verified in alcoholic liver disease and nonalcoholic fatty liver disease.^[10,11]^ On the other hand, short-chain fatty acids regulate the PPAR-α pathway through the GPR41/43 receptor and inhibit lipid synthesis mediated by SREBP1. Furthermore, short-chain fatty acids also reshape the Th17/Treg immune balance through the ERK1/2 signaling. This dual regulatory effect was clarified in the intervention studies of Dendrobium officinale and lactic acid bacteria.^[12,13]^

In the “intestinal leakage - Microbiota dysbiosis - liver injury” model, the key pathological links include: decreased expression of intestinal epithelial tight junction proteins, such as ZO-1 and occludin, leading to increased intestinal permeability, allowing microbiota products, such as LPS and ethanol metabolites, to enter the portal vein circulation^[11,14]^; production of secondary bile acids and aromatic hydrocarbon receptor ligands by the dysregulated microbiota, which interfere with liver lipid metabolism through the fanitol X receptor (FXR) pathway and simultaneously activate hepatic stellate cells to promote fibrosis^[10]^; the inflammatory microenvironment of the liver feedback affects the composition of the intestinal microbiota through changes in bile acid metabolism, forming a positive feedback loop.^[15]^ This cycle has been typically observed in both nonalcoholic fatty liver disease and alcoholic liver disease models, such as a decrease in Muribaculaceae bacteria in the intestines of mice on a high-fat diet accompanied by a 2.5-fold increase in serum LPS.^[11]^ Complete understanding of the significance of the hepaticogut axis in daily nursing requires close attention to the patients’ defecation status. Through daily inquiries and the defecation characteristics, such as stool volume, consistency, and rhythm (Table 2), we administered 15 mL of lactulose twice a day to soften the stool and 0.5 g of *Bacillus licheniae* twice a day to regulate the intestinal flora.^[16]^ Through drug intervention, the patients’ difficulty in defecation and stool dryness were effectively controlled. Meanwhile, the rhythm of defecation became regular, and the intestinal mucosal barrier was more effectively protected.^[14]^ Combined with the liver function indicators, we observed further improvements in liver function. From this, we found that the specific intervention of nursing can remodel the gut-liver axis function and further promote the recovery of liver function, this finding is also supported by recent studies conducted by scholars in the relevant field.^[17]^

### 3.2. Nutritional support

At present, the diagnosis and treatment methods for acute viral hepatitis E mainly include symptomatic treatment and supportive care. Due to the lack of specific drugs, the therapeutic effect is limited. Therefore, it is particularly necessary to explore more effective care and treatment strategies. This study adopted the case analysis method to systematically evaluate the disease progression of patients with acute icteric hepatitis E after ECMO following nutritional support care. In our case, the NRS2002 score of nutritional screening at the onset of the disease was 6 points. After being transferred to the gastroenterology department, the nutritional screening score was 2 points, promoting a consultation from the nutrition department. Considering the patient’s height of 163 cm and weight of 65 kg, a low-fat semiliquid diet was prescribed, with a total calorie count of 150 to 1600 kilocalories, including 75 to 80 g of protein rich in branched-chain amino acids, 40 to 45 g of fat, 200 to 225 g of carbohydrates. Additionally, the patient was supplemented with sufficient vitamins, minerals, and dietary fiber. The research results showed that nutritional support nursing intervention significantly improves the severity of jaundice and liver function indicators, especially playing an important role in enhancing the immune function and overall nutritional status of patients.^[18]^ These results indicate that nutritional support nursing has potential clinical application value in improving the prognosis of patients with acute icteric hepatitis E, providing a basis for optimizing related treatment plans.

**Figure 4.**
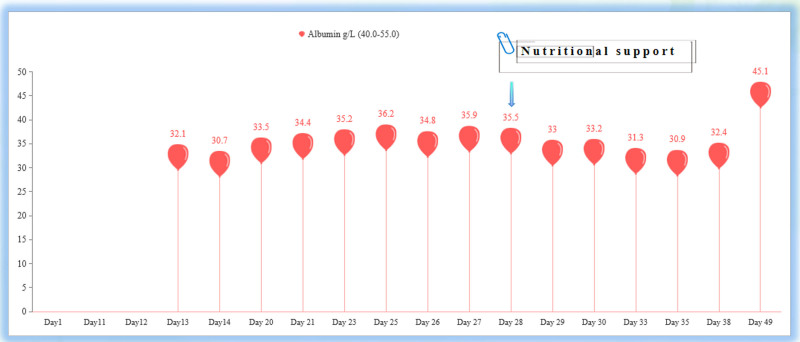
After nutritional intervention, serum albumin levels increased.

**Figure 5.**
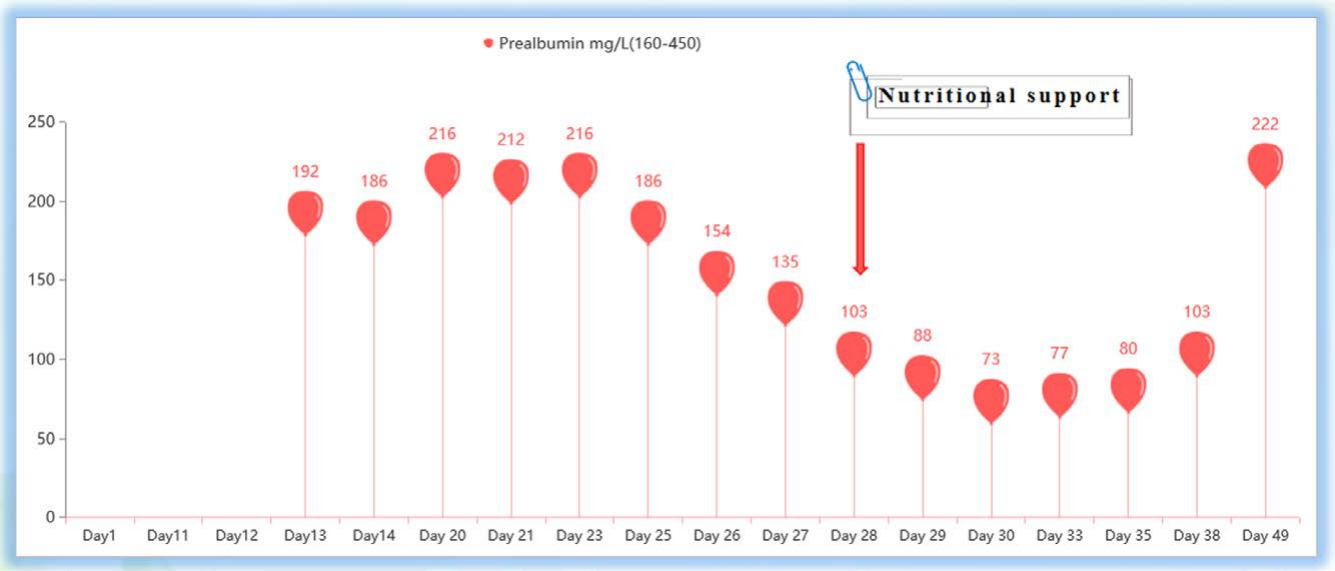
After nutritional intervention, serum prealbumin levels increased.

Studies have found that after patients received nutritional support, their liver function indicators, such as ALT and AST levels, decreased significantly. This suggests that nutritional intervention may significantly reduce total bilirubin levels by improving bile excretion, reducing inflammation, and promoting liver cell repair.^[19]^ This is consistent with our case. The relationship between nutritional support and the degree of jaundice is also worth exploring. We observed that under comprehensive diagnosis and treatment from the nutrition department, the nursing staff, and doctors, through blood biochemistry monitoring, the levels of serum albumin and prealbumin were found to gradually increase (Figs. 4 and 5). Unbound bilirubin and albumin were strongly combined to form bound bilirubin, and through the hepatoenteric circulation and renal metabolism, the level of serum bilirubin was significantly reduced. This indicates that nutritional intervention may help improve the metabolic function of the liver. Thereby alleviating the symptoms of jaundice. This finding is consistent with other studies, emphasizing the importance of good nutritional status for bilirubin metabolism.^[20]^

Additionally, nutritional support has been found to enhance the immune function of patients. Studies show that after nutritional support, immune-related indicators such as white blood cell count and immunoglobulin levels increase, suggesting that a good nutritional status may help enhance the patient’s immune capacity, thereby reducing the risk of infection^[21]^ and improving the recovery rate.^[22]^

Notably, individualized nutritional intervention programs have also shown a positive role in improving the overall rehabilitation effect of patients. According to the different nutritional needs of patients, the implementation of personalized nutrition plans has improved the overall rehabilitation effect of patients, especially in restoring liver function and improving the quality of life.^[23]^ Therefore, it is crucial to assess the nutritional needs of patients and formulate personalized nutritional support plans in clinical practice^[24]^ (Fig. 2).

Finally, nutritional support nursing intervention has also demonstrated economic benefits. Nutritional support is associated with shorter hospital stays and reduced medical expenses for patients (saving about 15%). This not only improves the treatment effect of patients but also effectively saves medical resources and enhances economic benefits.^[25]^ Therefore, nutritional support has significant clinical significance in the management of patients with acute icteric hepatitis E. Future studies should explore its specific application in the management of liver diseases.

#### 3.2.1. *Risks related to blood transfusion*

As one of the critical resuscitation measures, ECMO requires the infusion of a total of 8.5 U of red blood cells and 1810 mL of plasma into this patient before and after the treatment. While successfully resuscitating the patient, it also introduces risks related to blood transfusion. Studies have found that secondary acute hepatitis E after blood transfusion, especially in patients with compromised immune function, such as those with malignant tumors or organ transplants, is also associated with blood products.^[26–28]^ In this case, the patient experienced sudden cardiac arrest. During resuscitation, not only large doses of epinephrine and norepinephrine were needed to maintain hemodynamics, but methylprednisolone was also administered for anti-inflammatory treatment to suppress immune storm. Immunosuppressants can inhibit T cell function and weaken the virus-specific immune response during use.^[29]^ Studies have found that HEV infection can reduce CD8 + T cells and, in severe cases, lead to T cell exhaustion.^[30]^ Considering the influence of multiple factors, critically ill patients with weakened immunity have a significantly increased risk of secondary HEV infection during blood transfusion. Therefore, when blood transfusion patients experience fatigue, poor appetite, and yellowish skin and urine, it is essential to strengthen liver function tests and remain vigilant about the possibility of secondary hepatitis E virus from blood transfusion. When conditions permit, sample testing of blood donors can be strengthened for traceability, thus reducing the possibility of secondary infections due to blood transfusion.

In conclusion, this case analysis demonstrates that in addition to strengthening the monitoring of patients’ clinical symptoms and maintaining the balance of intestinal microecology, it is also necessary to enhance nutritional support, closely observe the mental health of patients, promptly guide and relieve tension and anxiety, and strengthen health education related to hepatitis E disease. These nursing interventions have had a significant impact on the prognosis of patients with acute icteric hepatitis E after ECMO, demonstrating positive effects in improving liver function and the severity of jaundice. This study has various limitations in practical work, which may affect the breadth and depth of the research results. Firstly, the single-case design is a core limitation. The study was conducted based on the diagnosis and treatment process of only one 35-year-old female patient, with an extremely small sample size, which greatly restricts the generalizability of the research conclusions. Patients of different ages, genders, and with underlying diseases may have different responses to hepatitis E infection and different effects of nursing interventions, so the experience from this case is difficult to be directly extended to a wider range of patient groups. Secondly, there is a lack of monitoring of key data. The study did not perform quantitative detection of HEV RNA, while the level of HEV RNA is an important indicator for evaluating viral replication activity and infection progress. Its absence may affect the accuracy of judging the severity of infection, treatment response, and prognosis, and also limits the in-depth analysis of the mechanism of transfusion-transmitted HEV. Furthermore, the follow-up time is relatively short. Although the 3-month postoperative follow-up showed no signs of chronic liver damage, the short-term observation of 3 months may fail to capture long-term prognosis, such as potential liver fibrosis, virus recurrence, or long-term impact on cardiac function, making it difficult to comprehensively evaluate the long-term effectiveness of the intervention measures. In addition, there are obstacles in blood source tracing. Since routine HEV screening is not carried out in blood banks in this region, it is impossible to perform HEV RNA detection on the 8.5 U red blood cell suspension and 1810 ml plasma transfused to the patient to identify the source of infection. This makes the demonstration of the causal relationship between transfusion-transmitted HEV lack direct etiological evidence support, increasing the uncertainty of the conclusion. Finally, the quantitative evaluation of nursing interventions is insufficient. Although the study mentions nursing measures such as intestinal flora regulation and nutritional support, it does not conduct quantitative analysis on the intervention intensity (such as the basis for adjusting the dose of probiotics), implementation details (such as the dynamic optimization process of the nutritional plan), and the independent effects of various measures. It is thus difficult to clarify the specific contribution of different nursing strategies to the patient’s recovery, which affects the standardization and promotion of the nursing plan. These limitations suggest that future studies need to expand the sample size, extend the follow-up period, improve virological monitoring and blood source tracing mechanisms, and strengthen the quantitative evaluation of nursing interventions to enhance the scientificity and clinical application value of the research.

We sincerely thank Junjun Wu for verifying the data and managing the patients, Wenli Wu for collecting the data, Dr Zheng for providing nutritional guidance to the patients, and Lili Dong for reviewing and organizing the content and ideas of the article. We also extend our gratitude to the department for their support of this research. The writing of this paper was completed independently by the author.

## Acknowledgments

We sincerely thank Junjun Wu for verifying the data and managing patient-related affairs, Wenli Wu for data collection, Su Zheng for providing nutritional guidance to patients, and Lili Dong for reviewing and organizing the article’s content and ideas. We also extend our gratitude to the Department of Internal Medicine for supporting this research. The writing of this paper was completed independently by the authors.

## Author contributions

**Conceptualization:** Lili Dong.

**Data curation:** Junjun Wu, Fang Ding, Wenli Wu.

**Formal analysis:** Rong Xu, Fang Ding.

**Investigation:** Rong Xu, Junjun Wu, Wenli Wu.

**Methodology:** Junjun Wu, Wenli Wu.

**Project administration:** Lili Dong.

**Software:** Fang Ding.

**Supervision:** Lili Dong.

**Writing – original draft:** Rong Xu.

**Writing – review & editing:** Lili Dong.
